# Association of Liver Function Tests With the Severity and Outcome of Dengue Fever in Children

**DOI:** 10.7759/cureus.67700

**Published:** 2024-08-24

**Authors:** Om Prasanth Reddy Avuthu, Ambrish Mishra, Manojkumar G Patil, Balasubramanya S Tandur, Shradha Salunkhe, Gaurav Kumar, Kasireddy Sravanthi, Srinija Garlapati, Shailaja V Mane, Pramod Jog

**Affiliations:** 1 Pediatrics, Dr. D. Y. Patil Medical College, Hospital and Research Centre, Dr. D. Y. Patil Vidyapeeth (Deemed to be University), Pune, IND; 2 Family Medicine, Dr. D. Y. Patil Medical College, Hospital and Research Centre, Dr. D. Y. Patil Vidyapeeth (Deemed to be University), Pune, IND

**Keywords:** vector-borne diseases, viral fever, liver function tests (lfts), dengue fever/complications, pediatrics & neonatology

## Abstract

Background

Dengue is one of the most common vector-borne diseases in India, and it is transmitted by *Aedes* family mosquitoes. Hepatic injury is known to occur from dengue infection. Direct hepatotoxicity and deranged host immune responses to the virus are responsible for this hepatic dysfunction. Hence, the study was undertaken to understand the deranged hepatic enzymes using liver function tests (LFTs) and the severity and outcome of dengue fever in children.

Methods

This study is an observational-descriptive study conducted between June 2022 and May 2024. The study population includes children between the ages of one month and 16 years who have been diagnosed with dengue fever and admitted to pediatric wards and pediatric intensive care units (PICUs), with a sample size of 151. Informed consent from guardians and institutional ethical clearance were obtained.

Results

A total of 4.8% (N = 7) mortality was seen in this study with dengue patients. Hepatomegaly was seen in 34% (N = 49) of cases. There is a clear statistical significance that is seen among the non-survived and survived dengue patients with a 10-fold increase in serum glutamic-oxaloacetic transaminase (SGOT) and serum glutamic pyruvic transaminase (SGPT) levels, respectively, along with total bilirubin, activated partial thromboplastin time (APTT), and prothrombin time (PT).

Conclusions

The current study shows that deranged LFTs are associated with more severe disease with more PICU admissions and mortality of the disease. The evidence clearly indicates the inclusion of LFTs as a routine investigation to understand the severity of the disease and the prognosis of the outcome.

## Introduction

Dengue is a common mosquito-borne disease that predominantly affects tropical and subtropical regions, including South and Southeast Asia. It is caused by the dengue virus, a single-stranded RNA virus, and is transmitted by *Aedes* mosquitoes [1]. According to global disease estimates, between 1990 and 2021, the incidence of dengue cases increased from 23.33 million to 58.86. million worldwide [2]. By 2019, dengue outbreaks had spread to 129 countries, marking the peak year for reported instances [3]. It is estimated that approximately 390 million dengue infections happen every year in the endemic regions, with a death toll reaching more than 20,000 cases annually [4].

Dengue presents in humans with a wide range of clinical spectrums, including both symptomatic and asymptomatic clinical manifestations. The World Health Organization (WHO) categorizes dengue as dengue with or without warning signs and severe dengue. Severe dengue is defined by the presence of dengue fever along with any of the following: severe plasma leakage leading to shock, severe hemorrhage, or organ failure [5].

Warning signs included a wide range of clinical symptoms such as severe abdominal pain, persistent vomiting, bleeding manifestations (mucosal bleeding, petechiae, ecchymosis), lethargy, hepatomegaly (enlarged liver), and other blood parameters such as hemoconcentration, indicated by increasing hematocrit levels and a rapid decline in platelet count [5,6].

It is to be noted that even though dengue is a non-hepatotropic virus, hepatomegaly is one such manifestation that is seen along with an increase in serum aminotransferases. Liver dysfunction seen in dengue infection may be caused by a direct attack of the virus on liver cells or by an impaired host immune system damaging the liver cells. The dysfunction can range from mild hepatic injury to fulminant hepatic failure [7].

Hence, hepatic manifestations in dengue need to be examined with liver function tests (LFTs) to understand the morbidity and mortality of the dengue infection, including the outcome of the disease [8,9]. However, there is a lack of clinical studies relating liver function and dengue severity to outcomes, especially in children. Hence, the study was conducted to understand the deranged hepatic enzymes using LFTs and the severity and outcome of dengue fever in children.

## Materials and methods

Study design and setting

This study is an observational cross-sectional study conducted on the hospitalized patients of the Department of Pediatrics at Dr. D. Y. Patil Medical College, Hospital, and Research Centre (DYPMCH), Pune, Maharashtra, India, from August 2022 to July 2024. The study received institutional ethical clearance from the DYPMCH (approval number IESC/PGS/2022/29, dated November 12, 2022).

Sample size

With reference to the study by Wilder-Smith and Rupali [10], considering the seroprevalence of dengue in children as 49% at a 95% confidence interval and an acceptable absolute error of 8%, the minimum sample size was calculated to be 151. The software used was WinPepi v11.64.

Inclusion criteria

This study included children aged one month to 16 years who presented with fever and were NS1 antigen-positive for dengue, positive for IgM and IgG antibodies against the dengue virus in the laboratory, and admitted to the DYPMCH hospital.

Exclusion criteria

Any clinical febrile illness other than dengue infection and those who are not willing to give written informed consent are excluded from the study.

Informed consent

Informed consent was obtained from the parents or legal guardians of each patient before enrolling in the study. For adolescents above 13 years old (male and female), assent was also obtained before participating in the study.

Data collection

The data were collected in detail using a structured proforma, which includes the following: (1) history of symptoms, such as detailed documentation of the patient's symptoms such as fever, muscle pain, joint pain, rash, and other signs suggestive of dengue; (2) clinical examination findings, including observations and physical examination results, recorded by healthcare professionals. This includes vital signs, physical manifestations such as rashes, bleeding, signs of dehydration, or other abnormalities; (3) laboratory test results confirming dengue infection, which typically involve serological tests (such as NS1 antigen, IgM, and IgG antibodies), or molecular tests (such as RT-PCR). It would also include other relevant lab results, such as a complete blood count (CBC), platelet counts, and LFT. The patients were managed according to WHO dengue management protocol; and (4) the final health status of the patient, including recovery, complications, or mortality.

Statistical analysis

Data were entered into a Microsoft Excel 2010 (Microsoft Corporation, Redmond, Washington) spreadsheet and imported into IBM SPSS Statistics for Windows, Version 20 (Released 2011; IBM Corp., Armonk, New York) for the analysis. Descriptive statistics include percentages, averages, and standard deviations. The chi-square test or Fisher's exact test assessed the statistical significance of the categorical variables. Student's t-test or Mann-Whitney U test was performed after assessing for normality using the Shapiro-Wilk test for assessing continuous numerical variables. A p-value of <0.05 was considered statistically significant for the parameters used.

## Results

Distribution of age among the study population

The mean age for participants who did not survive was 3.25 years (±4.61), and for those who survived, it was 6.41 years (±4.16). The school-going age group (n = 66) was found to be affected by the disease, followed by preschool (n = 29) and infants (n = 28). It was also observed that the survival rate is highest among school-going children with 66 (45.8%) cases, and the non-survival rate is highest among infants with three (42.8%) cases. The p-value of 0.631 indicates no statistically significant difference in age among the two outcome groups (Table 1).

**Table 1:** Distribution of age among the study population Fisher's exact p-value was used to calculate the statistical significance

S. No.	Variable	Non-survived n (%)	Survived n (%)	p-value
1	Infant (1 month–1 year)	3 (42.8)	25 (17.3)	0.631
2	Toddler (1–3 years)	1 (14.3)	19 (13.2)	
3	Preschool (3–5 years)	2 (28.6)	27 (18.8)	
4	School-going child (5–12 years)	0 (0)	66 (45.8)	
5	Adolescents (12–18 years)	1 (14.3)	7 (4.9)	
Total		7 (100)	144 (100)	

Distribution of sex among the study population

Among the non-survived patients, three (42.9%) were female and four (57.1%) were male. Among the survived patients, 58 (40.3%) were female, and 86 (59.7%) were male. The p-value obtained from Fisher's exact test is 1.0, which indicates that no statistically significant difference was found in the current study.

Distribution of symptomatology among the study population

Fever is a universal symptom presented in 151 (100%) of cases, whereas cough is presented in 51 (34.4%) cases, abdominal pain in 68 (45%), vomiting in 56 (37%), abdominal distension in eight (5.2%), decreased oral acceptance in 22 (14.5%), seizures in 14 (9.2%), increased work of breathing in 26 (17.2%), bleeding manifestation in 28 (18.5%), rashes in 66 (43.7%), and hepatomegaly in 49 (34%) cases. A significant association has been found between symptoms of abdominal distension, bleeding manifestations, hepatomegaly, and increased work of breathing (in the form of tachypnea, use of accessory respiration muscles) and patient survival (Table 2).

**Table 2:** Distribution of symptomatology among the study population Fisher's exact p-value was used to calculate the statistical significance

S. No.	Symptoms	Non-survived n (%)	Survived n (%)	p-value
1	Cough	3 (42.9)	49 (34.1)	0.692
2	Abdominal pain	4 (57.1)	64 (44.4)	0.701
3	Vomiting	3 (42.9)	53 (36.8)	0.711
4	Abdominal distention	2 (28.6)	6 (4.2)	0.045
5	Decreased oral acceptance	1 (14.3)	21 (14.6)	1.000
6	Seizures	2 (28.6)	12 (8.3)	0.128
7	Increased work of breathing	4 (57.1)	22 (15.3)	0.017
8	Bleeding manifestation	5 (71.4)	23 (15.9)	0.003
9	Rashes	5 (71.4)	61 (42.4)	0.241
10	Hepatomegaly	5 (71.4)	44 (30.7)	0.037
Total		7 (100)	144 (100)	

Distribution of the severity of the disease among the study population

Among those who did not survive, three (42.8%) had dengue with hemophagocytic lymphohistiocytosis (HLH), which is the highest. Interestingly, zero deaths are observed in cases of dengue fever without warning signs and dengue with hepatitis (Table 3).

**Table 3:** Distribution of the severity of the disease among the study population

S. No.	Diagnosis	Non-survived n (%)	Survived n (%)
1	Dengue fever with warning signs	1(14.3)	71 (49.3)
2	Dengue fever without warning signs	0	42 (29.2)
3	Dengue encephalitis	1 (14.3)	8 (5.5)
4	Dengue with hemophagocytic lymphohistiocytosis (HLH)	3 (42.8)	2 (1.4)
5	Dengue with hepatitis	0	6 (4.2)
6	Dengue shock	2 (28.6)	15 (10.42)
Total		7 (100)	144 (100)

Distribution of laboratory parameters among the study population

Significant differences were observed in the values of hemoglobin (Hb), total leukocyte count (TLC), total bilirubin, serum glutamic-oxaloacetic transaminase (SGOT), serum glutamic pyruvic transaminase (SGPT), activated partial thromboplastin time (APTT), and prothrombin time (PT) between those who did not survive and those who did. These differences suggest that higher levels of these parameters are associated with higher mortality in this context (Table 4).

**Table 4:** Distribution of laboratory parameters among the study population *The Student's t-test or Mann-Whitney U test was performed after assessing for normality using the Shapiro-Wilk test Hb: hemoglobin; TLC: total leukocyte count; SGOT: serum glutamic-oxaloacetic transaminase; SGPT: serum glutamic pyruvic transaminase; ALP: alkaline phosphatase; APTT: activated partial thromboplastin time; PT: prothrombin time; INR: international normalized ratio

Outcome	Non-survived (n = 7)		Survived (n = 144)		p-value*
Variable	Mean	Std. Deviation	Mean	Std. Deviation	
Hb	8.99	4.42	11.45	2.32	0.03
TLC	17142.86	13708.86	8381.54	7905.21	0.02
Platelets	48571.43	40298.29	142097.90	155759.13	0.13
Total bilirubin	3.54	6.88	0.58	0.64	<0.001
Direct bilirubin	0.61	0.73	0.51	2.20	0.98
SGOT	3333.57	6607.34	237.43	732.36	<0.001
SGPT	1493.14	2815.47	98.28	243.28	<0.001
ALP	184.00	104.43	191.37	124.09	0.98
APTT	61.46	40.06	31.04	8.25	<0.001
PT	26.00	12.85	12.93	5.26	<0.001
INR	2.36	1.13	1.38	1.59	0.27

Distribution of lab parameters with admission among the study participants

Regarding the bilirubin levels among ward admissions, 91 (97.8%) had normal levels, and two (2.2%) had elevated levels. Among pediatric intensive care unit (PICU) admissions, 50 (86.2%) had normal levels, and eight (13.8%) had elevated levels. For direct bilirubin levels, 47 PICU admissions (81%) had normal levels, and 11 (19%) had elevated levels. Among ward admissions, 90 participants (96.8%) had normal levels, and three (3.2%) had elevated levels. For SGOT levels, 12 PICU admissions (20.7%) had normal levels, and 46 (79.3%) had elevated levels. Among ward admissions, 46 participants (49.5%) had normal levels, and 47 (50.5%) had elevated levels. For SGPT levels, 31 (53.4%) had normal levels, and 27 (46.6%) had elevated levels in PICU admissions. Among ward admissions, 73 (78.5%) had normal levels, and 20 (21.5%) had elevated levels. For platelet counts, 47 PICU admissions (81%) had decreased levels, and 11 (19%) had normal levels. Among ward admissions, 58 participants (62.4%) had decreased levels, and 35 (37.6%) had normal levels (Table 5).

**Table 5:** Distribution of lab parameters with admission among the study participants *Fisher's exact p-value was used to calculate the statistical significance #The chi-square test p-value was used to calculate the statistical significance PICU: pediatric intensive care unit; SGOT: serum glutamic-oxaloacetic transaminase; SGPT: serum glutamic pyruvic transaminase

S. No.	Lab Parameters	PICU n (%)	Ward n (%)	p-value
1	Total bilirubin			
	Normal	50 (86.2)	91 (97.8)	0.014*
	Elevated	8 (13.8)	2 (2.2)	
	Total	58 (100)	93 (100)	
2	Direct bilirubin			
	Normal	47 (81)	90 (96.8)	0.003*
	Elevated	11 (19)	3 (3.2)	
	Total	58 (100)	93 (100)	
3	SGOT			
	Normal	12 (20.7)	46 (49.5)	<0.001#
	Elevated	46 (79.3)	47 (50.5)	
	Total	58 (100)	93 (100)	
4	SGPT			
	Normal	31 (53.4)	73 (78.5)	<0.001#
	Elevated	27 (46.6)	20 (21.5)	
	Total	58 (100)	93 (100)	
5	Platelets			
	Decreased	47 (81)	58 (62.4)	<0.01#
	Normal	11 (19)	35 (37.6)	
	Total	58 (100)	93 (100)	

## Discussion

In the present study, of the total 151 cases, 18.5% are infants, 13.2% are toddlers, 19.2% are preschool children, 43.7% are schoolchildren (the majority in this study), and 5.2% are adolescents. This finding is consistent with that of Anand et al. [11], who also reported a substantial proportion of cases in children aged 5-14 years. In contrast, Kumawat et al. [12] observed peaks in both children and young adults (15-29 years), indicating variability in age distribution across different studies and regions. This variability suggests that age-related susceptibility to dengue may be influenced by factors such as immune status, exposure patterns, and possibly variations in circulating viral strains. No association between the age group and mortality was observed in this study.

The present study identified a slight male predominance among dengue cases, with 56% of cases being male. This finding closely aligns with Anand et al.'s [11] findings of 54% male cases and Kumawat et al.'s [12] report of 57% male cases. In contrast, Mishra et al. [13] noted a more balanced sex distribution. The consistent male preponderance observed across multiple studies suggests a potential biological or behavioral factor influencing dengue susceptibility in males.

Fever was universally present in all dengue cases in this study, highlighting its diagnostic importance. Vomiting (52%) and abdominal pain (48%) were the most reported symptoms, replicating the findings reported by Mishra et al. [13]. Anand et al. [11] reported a higher prevalence of rash (46%), whereas this study found rash in 30% of cases. These variations in symptom prevalence underscore regional differences in clinical presentation, possibly influenced by viral strain characteristics or host immune responses.

Fever was the most prevalent symptom, with vomiting and loss of appetite also commonly observed among our patients. Other frequently noted symptoms included joint pain, abdominal pain, and headache, consistent with findings from various studies such as Mishra et al. and Ramabhatta et al. [13,14]. Abdominal distention was found in 5% of cases, and increased work of breathing was seen in 17% of cases. An association between mortality and these symptoms has been observed in this study.

On examination, hepatomegaly was seen in 32.4% of cases, and the correlation between liver enzymes and the severity of the disease is clearly seen among the survived and non-survived patients. Among the survivors and non-survivors, there is a 15-fold increase in the SGOT and SGPT levels. This is followed by a three-fold increase in total bilirubin and a two-fold increase in PT and APTT. All these laboratory parameters also show a statistically significant result. This is similar to the study conducted by Raju et al. [15], where there is a 10-fold elevation of SGOT among survivors and non-survivors, and similar findings were followed by Chinna et al. [16].

A statistically significant association was found upon comparing the PICU and ward admissions on SGOT, SGPT, and bilirubin levels, indicating the importance of hepatomegaly and deranged LFTs leading to severe infection and understanding the severity of the dengue infection. This correlated to a study conducted by Swamy et al. [17], which reported that SGOT and SGPT levels were elevated in 73.3% and 50.8% of the patients, respectively; SGOT was elevated in 66.7% of patients with dengue without warning signs, 78.6% with warning signs, and 91.7% with severe dengue. SGPT was elevated in 42.4%, 52.4%, and 91.7% of the patients with dengue without warning signs, with warning signs, and severe dengue, respectively. The mean SGPT, ALP, SGOT, total bilirubin, and direct bilirubin were statistically significant and higher among severe dengue compared to dengue with warning signs and dengue without warning signs. Mishra et al. [13] also showed a significant correlation between hepatomegaly and elevated SGOT levels. Approximately 86.59% were classified as non-severe and 13.40% as severe dengue.

Our study has a few limitations. As a tertiary care hospital, severe cases with terminal stages of disease are referred to our hospital, which could influence our results. Additionally, equal representation of all age groups is not seen in our study, as adolescents aged 14 to 16 tend to visit general medicine outpatient departments (OPD) than pediatric OPD, resulting in their underrepresentation.

## Conclusions

This study aims to elucidate the association between dengue fever and hepatic involvement. The findings show that among dengue children, deranged LFTs are associated with more severe disease, with more PICU admissions and mortality. The evidence clearly indicates the inclusion of LFT as a routine investigation to understand the severity of the disease and the prognosis in children suffering from dengue fever.
